# Structural magnetic resonance imaging predictors of responsiveness to cognitive behaviour therapy in psychosis

**DOI:** 10.1016/j.schres.2009.08.007

**Published:** 2009-12

**Authors:** Preethi Premkumar, Dominic Fannon, Elizabeth Kuipers, Emmanuelle R. Peters, Ananatha P.P. Anilkumar, Andrew Simmons, Veena Kumari

**Affiliations:** a Department of Psychology, Institute of Psychiatry, King's College London, London, UK; b NIHR Biomedical Research Centre for Mental Health, South London and Maudsley NHS Trust, London, London, UK; c South London and Maudsley NHS Trust, London, UK; d Centre for Neuroimaging Sciences, Institute of Psychiatry, King's College London, London, UK

**Keywords:** Grey matter, Voxel-based morphometry, Cerebellum, Cognitive flexibility, Memory, Schizophrenia

## Abstract

**Background:**

Responsiveness to cognitive behaviour therapy (CBT) in psychosis may have a neurological basis. This study aimed to determine whether improvement in symptoms following CBT for psychosis (CBTp) in people with schizophrenia is positively associated with pre-therapy grey matter volume in brain regions involved in cognitive processing.

**Methods:**

Sixty outpatients stable on medication with at least one distressing symptom of schizophrenia and willing to receive CBTp in addition to their standard care (SC), and 25 healthy participants underwent magnetic resonance imaging. Subsequently, 30 patients received CBTp (CBTp+SC; 25 completers) for 6–8 months and 30 continued with their standard care (SC; 19 completers). Symptoms in all patients were assessed (blindly) at entry and follow-up.

**Results:**

The CBTp+SC and SC groups did not differ clinically at baseline, and only the CBTp+SC group showed improved symptoms at follow-up. Severity of baseline symptoms was not associated with CBTp responsiveness. Reduction with CBTp in positive symptoms was associated with greater right cerebellum (lobule VII) grey matter volume, in negative symptoms with left precentral gyrus and right inferior parietal lobule grey matter volumes, and in general psychopathology with greater right superior temporal gyrus, cuneus and cerebellum (Crus I) grey matter volumes. Grey matter volume in these brain areas did not correlate with the severity of baseline symptoms.

**Conclusion:**

Grey matter volume of the frontal, temporal, parietal and cerebellar areas that are known to be involved in the co-ordination of mental activity, cognitive flexibility, and verbal learning and memory predict responsiveness to CBTp in patients with psychosis.

## 1 Introduction

A number of randomised-controlled trials have established that cognitive-behaviour therapy (CBT) for psychosis is efficacious (Wykes et al., 2008). Efficacy is clearest for patients who are antipsychotic medication-resistant (Kuipers et al., 1997, Tarrier et al., 1998), with acute patients not benefiting as much from therapy (Garety et al., 2008). Most studies have targeted positive symptoms of psychosis, but benefits have also been found in a number of different areas, such as negative symptoms, depression and social functioning (Wykes et al., 2008). A meaningful clinical response to CBTp, however, is seen in only about 50% of patients with psychosis who receive it (Pfammatter et al., 2006, Wykes et al., 2008).

Predictors of response to CBT for psychosis (CBTp) remain unclear. In one study, cognitive flexibility about delusions predicted the effect of CBTp on delusional thinking (Garety et al., 1997). CBTp requires patients with psychosis to generate alternative explanations of their psychotic experiences (Kuipers et al., 2006). Patients whose cognitive flexibility is challenged may not be able to consider new ways of thinking or coping. It is thought that CBT operates at the level of the cortex and explicit processing of meaning (Gorman et al., 1989, van der Gaag, 2006). CBTp responsiveness thus may be influenced by the integrity of specific brain regions that are involved in complex top-down processing of information (Kumari, 2006, van der Gaag, 2006).

This study aimed to determine whether improvement in symptoms following CBTp in people with psychosis when added to their standard care (SC) is positively associated with pre-therapy grey matter volume (GMV). A number of brain regions are found to be structurally deficient, on average, in people with schizophrenia compared to healthy people (Honea et al., 2005, Shenton et al., 2001) and these deficits in turn relate to impairment in a range of cognitive domains, including cognitive flexibility, in schizophrenia (Antonova et al., 2004).

We hypothesized that greater improvement in symptoms following CBTp+SC would be associated with greater GMV at baseline (pre-CBTp) in the frontal lobes, given previously reported positive associations between frontal lobe GMV and executive functioning, including cognitive flexibility, in schizophrenia (Antonova et al., 2004). However, given the dearth of studies on both cognitive and MRI predictors of CBTp, we explored positive associations between GMV and CBTp responsiveness across the whole brain. We also explored negative associations between GMV across the whole brain and CBTp+SC responsiveness and did not hypothesize for GMV in any specific regions to show a negative association.

## 2 Materials and methods

### 2.1 Participants and design

Participants were 60 outpatients with a DSM-IV diagnosis of schizophrenia or schizoaffective disorder who were willing to receive CBTp in addition to their usual care, and 25 healthy participants. Of these, 30 patients received CBTp+SC (25 completers) and 30 patients received SC (19 completers). The groups were matched on average for age, years in education and sex (Table 1). No participant had a history of neurological or organic illness or head injury.

**Table 1:** Demographic and clinical characteristics of study groups.

Characteristic	CBTp+SC patients (*n* = 25)		SC patients (*n* = 19)		Healthy participants (*n* = 25)	Model	*χ*^2^ (*df*)	*p*
Sex: male/female (*n*)	17/8		15/4		17/8	Group	0.80 (2)	0.672
							*F* (*df*)	
Age in years − mean, s.d.	35.6, 8.1		40.6, 9.8		33.7, 12.6	Group	2.48 (2,66)	0.090
Years in education − mean, s.d.	13.9, 3.1		13.5, 1.6		15.1, 2.5	Group	2.53 (2,66)	0.093
Diagnosis Schizophrenia/schizoaffective disorder	24/1		17/2		–	Group	0.72 (1,44)	0.395
Duration of illness a, years − mean, s.d.	11.1, 7.9		15.2, 11.5		–	Group	1.94 (1,42)	0.170
Age at illness onset, years − mean, s.d.	24.4, 8.0		25.4, 8.2		–	Group	0.16 (1,42)	0.691
Symptoms (PANSS b) − mean, s.d.	Baseline	Follow-up	Baseline	Follow-up				
Positive	18.1, 4.8	^▼^14.8, 4.2	18.4, 3.4	18.0, 3.8	–	Time × Group	5.84 (1,42)	0.024
Negative	17.9, 4.4	^▼^15.5, 4.2	19.2, 3.9	20.1, 4.6	–	Time × Group	6.67 (1,42)	0.016
General psychopathology	33.2, 7.2	^▼^28.3, 7.2	34.6, 4.6	34.3, 6.9	–	Time × Group	4.37 (1,42)	0.042
Total	69.1, 13.6	^▼^58.6, 14.5	72.2, 9.0	72.3, 13.1	–	Time × Group	7.93 (1,42)	0.007
Medication level^c^ − mean dose, s.d.	520.7, 380.7		484.7, 345.0		–	Group	0.11 (2,66)	0.748
Total grey matter volume (ml) − mean, s.d.	702.4, 72.0		697.5, 68.4		736.4, 72.5	Group	2.08 (2,66)	0.133
Total white matter volume (ml) − mean, s.d.	410.1, 47.5		415.3, 41.7		417.8, 48.9	Group	0.18 (2,66)	0.838
Total cerebro-spinal fluid (ml) − mean, s.d.	525.6, 87.2		527.9, 61.5		531.0, 82.7	Group	0.03 (2,66)	0.971

^▼^Lower symptom scores (*p* < 0.01) at follow-up in the CBTp + SC, but not in the SC, group.

a Difference between the age at onset of psychotic symptoms (as reported by the patient and, where possible, confirmed with clinical records and other sources) and current age.

b PANSS: Positive and Negative Syndrome Scale.

c Medication level expressed in chlorpromazine equivalents.

Patients were recruited from the South London and Maudsley (SLAM) NHS Foundation Trust, were on stable doses of antipsychotic medication for at least two years and on the current antipsychotic drug for at least three months prior to taking part (86% on atypical antipsychotics), and had a score of ≥ 60 on the Positive and Negative Syndrome Scale (PANSS, Kay et al., 1987) and at least one persistent positive symptom (a score of 3 or above on at least one of the positive symptoms items of the PANSS, which they experienced as distressing). The patients in the CBTp+SC and SC groups were recruited from the same geographical area, were identified by local psychiatric consultants as suitable for CBTp, and wished to receive CBTp in addition to their usual care. Patients who were referred to and accepted for CBTp by the Psychological Interventions Clinic for Outpatients with Psychosis (PICuP), SLAM NHS Foundation Trust went into the CBTp+SC group. The researchers did not have a say in which of the patients might receive CBT at this specialist clinic. With the resources available to the SLAM NHS Foundation Trust at the time of this investigation, only a small number (about 10%) of eligible patients were offered CBT for psychosis. Others who matched demographically and clinically as much as possible those accepted for CBTp by the PICuP were studied as part of the SC group over the same interval as the CBTp+SC group patients. SC consisted of case management offered by the case management team for a particular geographical area. The team includes a dedicated care coordinator who sees the patient on a regular basis. Six-monthly care plan assessment reviews are carried out with a focus on recovery. There was no change in antipsychotic dosage over the follow-up period in either group.

The study was approved by the local ethics committee. All participants provided written informed consent to their participation and were compensated for their time and travel.

### 2.2 Cognitive behaviour therapy

Therapy followed the procedure developed by Fowler and colleagues (1995). Therapy was delivered weekly or fortnightly, as preferred by the patient, over an average of 16 individual one-hour sessions. Therapy lasted for 6–9 months, according to NICE guidelines (NICE, 2008). It was based on individualized formulations, and aimed to reduce distress and interference arising from psychotic symptoms, reduce depression, anxiety and hopelessness, and modify dysfunctional schemas when appropriate. Initial sessions focused on facilitating engagement in therapy. The therapist endeavored to build and maintain a good therapeutic relationship by taking a flexible approach that focused on the patient's needs (Kuipers et al., 1997). The therapists were qualified CBT practitioners, and were supervised by experienced clinical psychologists (EK, EP) with expertise in CBTp.

### 2.3 Clinical assessments

Clinical diagnosis was confirmed by the Structured Clinical Interview for DSM-IV (First et al., 2002). PANSS assessments (Kay et al., 1987) were performed on all patients before and after CBTp+SC by an experienced psychiatrist (DF) who was blind to whether or not a patient received CBTp in addition to their usual treatment. This psychiatrist had no role in patient recruitment or clinical management of any of the patients included in this investigation. Appointments for these assessments were made by another member of the research team. We have no evidence that blindness was broken during these assessments.

### 2.4 Magnetic resonance imaging acquisition

All participants underwent structural magnetic resonance imaging (MRI) at baseline. MRI scans were acquired using a 1.5 Tesla GE NV/i Signa system (General Electric, Milwaukee WI, USA) at the Maudsley Hospital, London. Initially, a series of sagittal fast gradient echo scout images were acquired. A 3-D inversion recovery prepared fast spoiled GRASS sequence was applied to the whole brain to obtain T1-weighted images in the axial plane with 1.5 mm contiguous sections (TR = 18 ms, TI = 450 ms, TE = 5.1 ms, flip angle = 20° with one data average and a 256 × 256 × 128 voxel matrix). Acquisition parameters were chosen using a sophisticated image contrast simulation (Simmons et al., 1996).

### 2.5 MRI pre-processing

Structural images were converted into ANALYZE format (ANALYZE software, BRU, Mayo Foundation, Rochester, MN) and pre-processed using Statistical Parametric Mapping (SPM2; http://www.fil.ion.ucl.ac.uk/spm), running in MATLAB 2006a (MathWorks, Natick, MA) as previously described (Premkumar et al., 2008). Customised T1-weighted templates of the whole brain, grey matter, white matter and cerebro-spinal fluid (CSF) were created for patient and healthy participant groups separately, and also for the whole study sample (patients and healthy participants combined). For analyses concerning GMV correlates of symptom improvement in the CBTp+SC group, templates used were derived from the patient group alone. For comparisons between CBTp+SC responders, CBTp+SC non-responders and healthy participant groups, templates created for the whole study sample were utilized. Using a separate ‘patients only’ template for the analyses concerning GMV correlates of CBTp+SC allowed us to determine CBTp specific effects, without confounding for GMV-averaged effects of healthy participants.

### 2.6 Statistical analysis

#### 2.6.1 Baseline demographic and clinical characteristics and total brain volumes

Analyses of variance (ANOVA) were performed to examine baseline group differences (as applicable) in age, years in education, age of illness onset, duration of illness and medication. Gender distribution was examined using chi-square test.

The total grey matter, white matter and CSF volumes for each individual were calculated from the unsmoothed modulated segmented images using SPM2 and the values representing the volume in milliliters extracted. Intracranial volume was calculated as the sum of the grey matter, white matter and CSF volumes. Group differences in total grey matter, white matter and CSF volumes were examined in SPSS (v15) by means of ANOVA.

#### 2.6.2 Symptom change following CBTp

Repeated-measures ANOVAs were performed to determine symptom change from baseline to follow-up, with time (baseline, follow-up) as the within-subject factor and group (CBTp+SC and SC) as the between-subjects factor. Following significant symptom improvement at follow-up in the CBTp+SC group but not the SC group (see Results section), potential associations between symptom improvement and age, duration of illness, medication dosage (chlorpromazine equivalents) and baseline symptoms (total PANSS; positive, negative and general psychopathology subscale scores) were examined in the CBTp+SC group using Pearson's *r*. Such analyses were not performed in the SC group since this group did not show clinical change between the baseline and follow-up.

#### 2.6.3 MRI correlates of symptom change following CBTp

Analyses of GMV correlates of CBTp responsiveness were performed in SPM5 (5–1782). For the purpose of this analysis, treatment outcome in the CBTp+SC group, was estimated as the residual change in PANSS symptoms (Siegle et al., 2006). To examine the associations between baseline (pre-CBTp) GMV and symptom improvement in the CBTp+SC group, residual symptom change scores on the PANSS (total and three subscales) were regressed at each voxel across the whole brain. The resultant SPM correlation maps were thresholded at *p* < 0.005 uncorrected. Given the exploratory nature of this study and the suggestion that the procedure for correction for multiple comparisons in SPM (originally designed for the analysis of functional data) is overly strict when applied to structural data (Sowell et al., 1999), relationships between CBTp+SC responsiveness and brain regional volumes where clusters were larger than 300 voxels with a local maxima reaching *T* > 2.81 (*p* < 0.005) were treated as being of interest and a small volume correction (SVC; 10 mm radius sphere centred on the maxima voxel) was applied to determine whether a cluster is significant (*p* = 0.05, family-wise error corrected) after correcting for multiple comparisons within a locally defined volume.

We then extracted the values representing the percentage of total grey/white matter volume under a smoothing kernel relative to the total grey/white matter volume for each participant in the patient group at the maxima voxels of all the regions where GM volumes were associated with symptom change and/or baseline symptoms in the CBTp+SC group. Partial correlations were performed between these values and symptom change controlling for intracranial volume. To determine whether these associations might also be present in the SC group, the GM intensities at each significant voxel were also extracted for the SC group and correlations performed between these values and symptom change. Finally, scatterplots were used to explore whether GMV correlates of symptom improvement in CBTp+SC patients receiving clozapine (*n* = 6) differed from those receiving other types of antipsychotics.

#### 2.6.4 Group differences in regional brain volumes

Group differences in GMV were also examined in SPM5 (5–1782). We compared regional GMVs across the whole brain of CBTp+SC responders (patients who showed a 20% improvement from baseline on PANSS total symptoms) with that of CBTp+SC non-responders and healthy participants using independent sample *t*-tests and applied the same significance criteria as noted earlier for association with responsiveness to CBTp. Following the observation of group differences in hippocampal GMV, group differences were re-evaluated using masks for left and right hippocampus from the PickAtlas software (Maldjian et al., 2003).

## 3 Results

### 3.1 Baseline demographic and clinical characteristics and total brain volumes

The demographic and clinical characteristics and total grey matter, white matter and CSF volumes of the patients and healthy participants are presented in Table 1. The CBTp+SC and SC patient groups did not differ in demographic characteristics, symptoms, medication level, or brain variables at baseline.

### 3.2 Symptom change following CBTp

Symptom severity in the CBTp+SC group was reduced at follow-up relative to the SC group (Table 1). CBTp+SC patients showed an improvement, on average, of 14.5% (s.d. = 16.9%) on PANSS total symptoms following CBTp+SC, while SC patients showed no change. Post-CBTp improvement in symptoms was not associated significantly with any baseline clinical characteristic.

### 3.3 MRI correlates of symptom improvement following CBTp

**Fig. 1:**
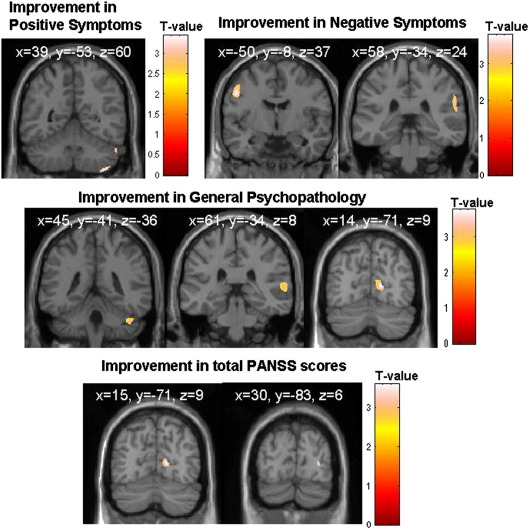
Grey matter areas (maps thresholded at *p* = 0.005 uncorrected) showing a positive association with symptom improvement following CBTp in patients.

In the CBTp+SC group, greater GMV in the right cerebellum was associated with improvement in positive symptoms (Table 2 and Fig. 1). Greater GMV in the left precentral gyrus and right inferior parietal lobule was associated with improvement in negative symptoms. Greater GMV in the right STG, cuneus and cerebellum was associated with improvement in general psychopathology. Greater GMV in the right cuneus and right STG was positively associated with improvement in total symptoms. These associations in the CBTp+SC group remained significant after controlling for intracranial volume, and were not found in the SC group (Table 3). There were no negative associations between GMV in any brain areas and symptom improvement.

**Table 2:** GMV correlates (positive associations) of symptom improvement following CBTp (*n* = 25) (height threshold *p* = 0.005 for all voxels; *T* > 2.81; family-wise error corrected *p* < 0.05).

Brain region	Side	BA^a^	MNI^b^ coordinates			Voxel *T* value	Number of contiguous voxels	Voxel equiv *Z*
			*x*	*y*	*Z*			
*PANSS positive symptoms improvement*								
Cerebellum (lobule VII)	Right		39	−52	− 59	3.21	313	2.89

*PANSS negative symptoms improvement*								
Precentral gyrus	Left	6	− 50	− 8	37	3.75	1244	3.29
Inferior parietal lobule	Right	40	58	− 34	24	3.72	1100	3.26

*PANSS general psychopathology improvement*								
Superior temporal gyrus	Right	22	61	− 34	8	3.8	1747	3.31
Cuneus	Right	17	14	− 71	9	3.86	708	3.35
Cerebellum (Crus I)	Right		45	− 41	− 36	3.83	955	3.33

*PANSS total symptoms improvement*								
Cuneus	Right	17	16	− 71	9	3.44	319	3.06
Superior temporal gyrus	Right	22	60	− 33	13	3.57	1662	3.15

a Broadmann area.

b Montreal Neurological Institute.

**Table 3:** Correlations between localized GMVs (with positive associations with CBTp response within SPM5) and symptom improvement in CBTp+SC and SC groups.

Brain region-symptom improvement correlate	CBTp+SC	CBTp+SC controlling for intracranial volume	SC
	*r* (*p*)	Partial *r* (*p*)	*r* (*p*)
Right cerebellum-positive symptoms	0.557 (0.004)	0.581 (0.003)	0.421 (0.073)
Left precentral gyrus-negative symptoms	0.615 (0.001)	0.653 (0.001)	− 0.315 (0.189)
Right inferior parietal lobule-negative symptoms	0.613 (0.001)	0.628 (0.001)	0.002 (0.994)
Right superior temporal gyrus-general psychopathology	0.621 (0.001)	0.676 (<0.001)	− 0.264 (0.275)
Right cuneus-general psychopathology	0.627 (0.001)	0.700 (<0.001)	0.216 (0.375)
Right cerebellum-general psychopathology	0.435 (0.030)	0.627 (0.001)	0.226 (0.352)
Right cuneus-total symptoms	0.583 (0.002)	0.618 (0.001)	0.267 (0.270)
Right superior temporal gyrus-total symptoms	0.597 (0.002)	0.654 (0.007)	− 0.288 (0.232)

**Fig. 2:**
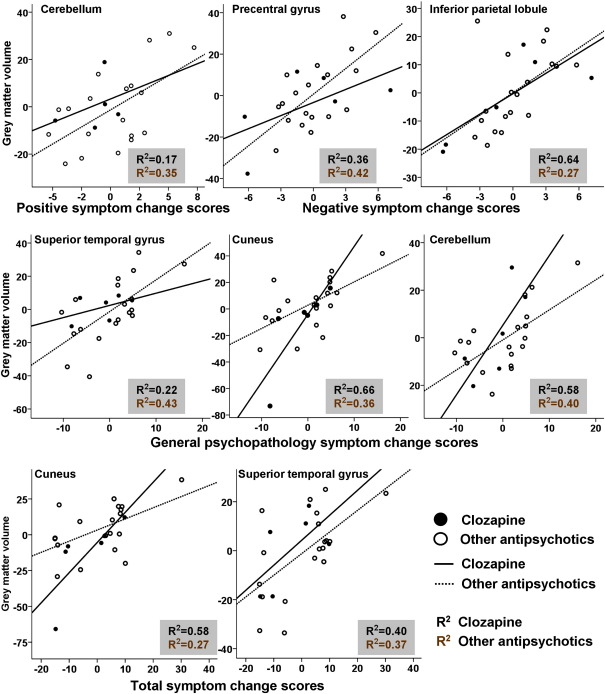
Scatter plots showing the relationship between the percentage grey matter volumes (relative to group mean) at the maxima voxel of all the regions (*y*-axis) showing an association with symptom change (*x*-axis) for CBTp+SC patients receiving clozapine or other antipsychotics.

The GMV correlates of post-CBTp symptom improvement appeared to be similarly present in patients receiving clozapine and those receiving other antipsychotics (Fig. 2).

### 3.4 Group differences in regional brain volumes

**Fig. 3:**
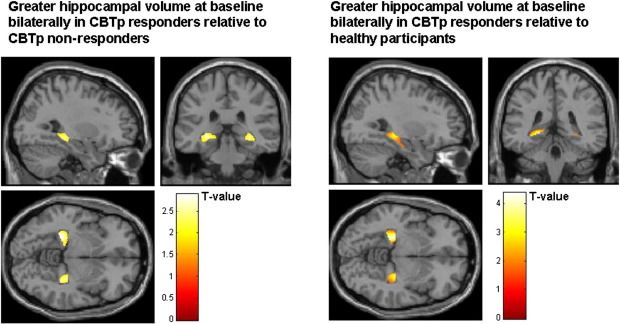
Greater posterior hippocampal volume (maps thresholded at *p* = 0.05 uncorrected) in CBTp+SC responders relative to healthy participants and CBTp+SC non-responders.

CBTp+SC responders (*n* = 11) had greater left and right hippocampal volumes relative to healthy participants and greater left hippocampal volumes relative to CBTp+SC non-responders (Table 4 and Fig. 3). No other group differences met the significance criteria.

**Table 4:** Differences in the posterior hippocampus GMVs between CBTp+SC responders (*n* = 11), CBTp+SC non-responders (*n* = 14) and healthy participants (*n* = 25) (height threshold *p* = 0.005, cluster size > 10 voxels).

Side	MNI^a^ coordinates			Voxel *T* value	Number of contiguous voxels	Voxel equiv *Z*
	*x*	*y*	*z*			
*CBTp+SC responders > healthy participants*						
Right	28	− 28	− 5	3.33	132	3.07
Left	− 25	− 30	− 5	4.36	797	3.86

*CBTp+SC responders > CBTp+SC non-responders*						
Left	− 27	− 36	− 5	2.89	11	2.64

a Montreal Neurological Institute.

## 4 Discussion

This study examined structural MRI predictors of symptom change following CBTp in patients with psychosis and found that the patients who received CBTp added to their usual care (SC), on average, showed improved symptoms at follow-up; SC patients showed the same level of symptoms at study entry and follow-up. In the CBTp+SC group, greater symptom improvement was associated with greater GMV as follows: (a) positive symptoms with right cerebellum (lobule VII), (b) negative symptoms with left precentral gyrus and right inferior parietal lobule, (c) general psychopathology with right STG, right cuneus and right cerebellum (Crus I), and (d) total symptoms with right STG and right cuneus. These associations were absent in the SC group. CBTp+SC responders (*n* = 11/25) had greater posterior hippocampal volume bilaterally relative to healthy participants and greater left posterior hippocampal volume than CBTp+SC non-responders.

### 4.1 MRI correlates of symptom improvement following CBTp

Contrary to our expectation, no significant association was found between CBTp responsiveness and prefrontal GMV. However, GMVs in a number of other regions as discussed below were positively associated with CBTp responsiveness.

We found that greater GMV in the right cerebellum (lobule VII and Crus I) was associated with improvement in positive symptoms and general psychopathology. Although the cerebellum, traditionally, has been implicated in motor control, recent data demonstrate cerebellar contributions to higher order cognitive functions, especially the task management and multi-tasking components of executive processing (Andreasen and Pierson, 2008), manipulation of information held in verbal working memory [in a manner similar to that observed for the prefrontal cortex (Hayter et al., 2007)] and co-ordination of mental activity (Thach, 2007), all of which are likely to be pertinent to effective CBTp. Patients with greater cerebellar GMVs may have been more capable of reasoning and relational processing (pooling together and comparing decision-relevant information) and benefited most from CBT. According to Andreasen and colleagues (Andreasen et al., 1999, Andreasen and Pierson, 2008), disruption in the cortico-cerebellar-thalamo-cortical circuitry results in deficient processing, prioritising, retrieval, coordination, and responding to information in schizophrenia. It is likely that functions of this circuitry are also relevant to CBTp responsiveness in schizophrenia as suggested previously (Kumari et al., 2009).

GMVs in the left precentral gyrus (BA6) and right inferior parietal lobule (BA40) were positively associated with improvement in negative symptoms. The precentral gyrus responds when information must be continuously updated and memory for temporal order maintained in working memory (Wager and Smith, 2003), while the parietal lobe is involved in both working and episodic memory (Olson and Berryhill, 2009). Functional imaging studies also reveal inferior parietal lobule involvement in sensory integration, body image, concept of self, and executive function in schizophrenia (Torrey, 2007). The association between larger fronto-parietal GMV and greater responsiveness to CBTp in this study could be mediated by the cognitive functions associated with these brain regions.

Greater GMV in the right cuneus (BA17) was associated with post-CBTp improvement in overall symptoms and general psychopathology. The cuneus is also part of the cortico-cerebellar-thalamic-cortical circuit proposed (mentioned earlier) by Andreasen and colleagues (1999) within the context of cognitive dysmetria in schizophrenia and shows activity changes during recall of practiced verbal material (Crespo-Facorro et al., 1999). Greater availability of cuneal GMV in patients may benefit better recall of discussions during therapy and in turn their ability to carry out home assignments.

Greater GMV in the right posterior STG was associated with CBTp-led improvement in total symptoms and general psychopathology. Greater bilateral posterior STG GMV has been associated with better abstraction (Nestor et al., 1993), and right posterior STG with fewer perseverative errors on the Wisconsin Card Sorting test (Nestor et al., 2007b) and good insight in schizophrenia (Cooke et al., 2008). Greater right posterior STG GMV thus may enable better cognitive flexibility and insight into their symptoms, and in turn facilitate CBTp responsiveness.

It is important to highlight that GMV alterations in certain brain areas in schizophrenia are associated with both low level of certain cognitive functions (Antonova et al., 2004) and severity of symptoms (review, Kumari and Cooke, 2006). We chose to consider the GMV correlates of CBTp responsiveness in terms of their neuropsychological significance because (a) there is no evidence to our knowledge (from any previous study or this study) that baseline symptom severity moderates the effects of CBT for psychosis, and (b) the GMVs associated with CBTp-led improvement were also not associated with baseline symptoms in this study. Furthermore, for the purpose of detecting relationships between pre-therapy GMVs and CBTp-led improvement, CBTp responsiveness was defined as residual change scores (estimated from baseline symptom severity-predicted change scores) following the methods proposed for such studies by Siegle and colleagues (2006). Although at present there are very limited data on predictors of CBTp in schizophrenia, studies in other psychiatric disorders, such as depression and anxiety, have highlighted the importance of pretherapy level of cognitive functions as moderators of responsiveness to CBT (Julian and Mohr, 2006, Mohlman and Gorman, 2005) and this may be even more salient in patients with schizophrenia who, on average, show deficient performance across a range of neuropsychological tasks (Reichenberg and Harvey, 2007).

### 4.2 Group differences in hippocampal volume

CBTp+SC responders showed greater posterior hippocampal volume compared to healthy participants and CBTp+SC non-responders. The hippocampal volume is positively associated with verbal learning and memory in psychosis (Antonova et al., 2004, Nestor et al., 2007a, Rametti et al., 2007). Although hippocampal volume is generally smaller in patients with schizophrenia (Honea et al., 2005, Shenton et al., 2001), a recent study (Thoma et al., 2009) reported smaller anterior, but not posterior, hippocampal volumes in schizophrenia patients, compared to controls; this study further showed that the anterior hippocampal volume correlated negatively, while the posterior hippocampal volume correlated positively, with memory measures in the schizophrenia group. Based on these previous findings, it can be suggested that greater posterior hippocampal volume facilitated memory for verbal materials and thus was conducive for treatment response in CBTp+SC responders. In a recent study, MacQueen and colleagues (2008) found greater bilateral hippocampal volume at baseline in patients with major depression who remitted, compared to those who did not remit, after 8-weeks antidepressant therapy (MacQueen et al., 2008). They suggested that greater hippocampal volume may afford better cognitive (Leal-Galicia et al., 2008, Wais, 2008) and emotion processing (Dolcos et al., 2005, LaBar and Cabeza, 2006, Phelps, 2004, Viveros et al., 2007) in psychiatric patients and enable them to reformulate their experiences (symptoms) resulting in improved symptoms following treatment. It is also possible that CBTp responders of our study had other characteristics that were not measured but may be associated with both greater-than-normal posterior hippocampal GMVs and a beneficial response to CBTp. For example, the right hippocampus is also enlarged in people who meditate (Holzel et al., 2008, Luders et al., 2009). However, we had not expected to find a greater-than-normal posterior hippocampal GMV in CBTp responders; this finding needs to be replicated before being taken seriously.

### 4.3 Limitations

A limitation of the study is that although some specific predictions were made about the neural networks associated with symptom change in response to CBTp+SC, the analysis was exploratory in nature as this is the first study to our knowledge to examine the brain structural predictors of CBTp+SC and our findings are based on a small sample. It is possible that the GMV correlates of CBTp responsiveness were confounded by antipsychotic drug treatment. We, however, did not find any differences in the distribution of GMV correlates between patients receiving clozapine (*n* = 6) and patients receiving other antipsychotic drugs. We were not able to determine the effect of mood stabilizers on the GMV-symptom improvement associations due to the small number of CBTp+SC patients on mood stabilizers (*n* = 4). Finally, in the absence of pre-CBTp assessment of relevant cognitive functions, the proposed neuropsychological significance of MRI predictors of CBTp remains speculative.

### 4.4 Conclusions

In conclusion, greater GMV of the brain areas that sub-serve the co-ordination of mental activity, cognitive flexibility, and verbal learning and memory may predict a beneficial response to CBTp in patients with psychosis. These findings need to be replicated in relatively larger samples and supplemented with pre-CBTp assessment of relevant cognitive functions using sophisticated neuropsychological tests along with MRI to confirm the neuropsychological significance of MRI predictors in a direct manner.

## Role of funding source

The sponsors had no role in study design; in the collection, analysis and interpretation of data; in the writing of the report; or in the decision to submit the paper for publication.

## Contributors

Veena Kumari, Elizabeth Kuipers and Preethi Premkumar designed the study. Dominic Fannon and Ananatha PP Anilkumar performed the clinical diagnostic interviews and symptom ratings. Emmanuelle R Peters and Elizabeth Kuipers supervised cognitive behaviour therapy for psychosis. Andrew Simmons contributed to development of MRI protocol. Preethi Premkumar assisted with data collection, scored and data-based clinical measures, undertook statistical analysis, and wrote the first draft of the manuscript.

## Conflict of interest

The authors declare no conflict of interest.
